# Converting the maybes: Crucial for a successful COVID-19 vaccination strategy

**DOI:** 10.1371/journal.pone.0245907

**Published:** 2021-01-20

**Authors:** Katie Attwell, Joshua Lake, Joanne Sneddon, Paul Gerrans, Chris Blyth, Julie Lee

**Affiliations:** 1 School of Social Sciences, University of Western Australia, Perth, Western Australia, Australia; 2 Wesfarmers Centre of Vaccines and Infectious Diseases, Telethon Kids Institute and School of Medicine, University of Western Australia, Perth, Western Australia, Australia; 3 Centre for Human and Cultural Values, University of Western Australia, Perth, Western Australia, Australia; 4 Department of Paediatric Infectious Diseases, Perth Children’s Hospital, Perth, Western Australia, Australia; 5 Department of Microbiology, PathWest Laboratory Medicine WA, QEII Medical Centre, Perth, Western Australia, Australia; Faculty of Science, Ain Shams University (ASU), EGYPT

## Abstract

**Background:**

Broad community acceptance of a COVID-19 vaccination will be critical for effectively halting the spread of the virus. In this study, we focus on factors that differentiate those who are undecided from those who are either willing or unwilling to accept a prospective COVID-19 vaccine.

**Methods:**

An online survey in May 2020 assessed Australian adults’ willingness to receive a COVID-19 vaccine (yes, maybe, no). A multinomial logistical regression of responses (N = 1,313) was used to identify correlates of vaccine willingness between the three groups.

**Results:**

65% were willing to vaccinate, with 27% being in the ‘maybe’ category. Respondents were more likely to be in the ‘maybe’ than the ‘yes’ group when they perceived COVID-19 to be less severe, had less trust in science, were less willing to vaccinate for influenza, and were female. They were more likely to be in the ‘maybe’ than ‘no’ group when they perceived COVID-19 as severe, and less likely to be a hoax, had more trust in science, and greater willingness to vaccinate for influenza. A repeat of the survey in November 2020 with a subset of participants found fewer of them saying yes to the vaccine (56%) and more saying maybe (31%).

**Conclusions:**

The effectiveness of any COVID-19 vaccine rollout will be reliant on maximizing uptake. The significant number of people who remain undecided about whether or not to get a COVID-19 vaccine, despite the ongoing devastating consequences of the virus for individuals, communities, and economies, is concerning. Our findings aid current research seeking to inform policy regarding how to convince the undecided to vaccinate.

## Introduction

Countries continue to fight devastating first and second waves of COVID-19 infections. Australia has, seemingly, just negotiated a challenging second wave. As communities and governments grapple with reintroducing lockdowns and ongoing economic uncertainty, a COVID-19 vaccine offers the best chance of restoring societal function. The World Health Organisation has identified 48 vaccine candidates currently in clinical evaluation [1]. Scholars of vaccine hesitancy and uptake are advising governments and the scientific community that an effective vaccine is just the beginning. Based on previous experiences with pandemic vaccines and vaccine hesitancy more generally, a well-researched strategy for rollout and acceptance will be necessary in every country.

Australia has agreements with four potential suppliers of different COVID-19 vaccines, and has invested in doses for its entire population [2]. Australians are highly supportive of vaccination in general, with recent studies finding that 87% of the population believe vaccines to be safe, effective, and necessary [3]; only 5.6% of Australians do not believe vaccines are safe [4]. However, most recent pandemic experience–the H1N1 outbreak in 2009, saw low vaccine uptake amongst adults, one study finding that 26% of refusers were concerned about safety and 17% did not believe in the vaccine [5].

Recent studies globally have reported relatively high acceptance rates for a COVID-19 vaccine (e.g., around 74%-77% in France [6], and 67–69% in the United States [7, 8]). In a comprehensive survey of 19 countries, 72% of participants indicated they were either likely or very likely to take a vaccine, ranging from 89% in China, to 55% in Russia [9]. Concerningly, however, studies conducted in Australia indicate a potential drop in support over time, with one study in April 2020 finding 86% of respondents willing to vaccinate [10], and a separate study in June finding 75% willing to vaccinate [11].

Importantly, most recent COVID vaccine studies have not examined the undecided (i.e., instead collapsing response scale points into positive and negative categories or using ‘indifferent’ or ‘no opinion’ as the mid- point on a yes/no scale). Even those that have collected data on those who are undecided [11] did not treat this group as the subject of analysis. Those who are currently undecided are likely to be substantial in number and crucial to the success of an effective COVID-19 vaccination program both locally and internationally. The present study examined the acceptability of a COVID-19 vaccine among a national sample of older Australian adults, with a focus on those who were uncertain about whether or not they would accept a COVID-19 vaccination.

The onset of COVID-19 in Australia converged with the onset of influenza season, leading to government authorities and health professionals making strong recommendations for Australians to not delay their seasonal vaccines [12]. Limiting the numbers of influenza cases would protect hospitals’ surge capacity, and reduce incidences of misdiagnoses, quarantine, and testing [13]. Take-up was vigorous, as influenza vaccines are nationally funded for at-risk groups, including children, and affordable for others through workplace clinics and community pharmacies, as well as being available through General Practitioners [12]. Our study also explored intentions regarding influenza vaccination in a context of COVID-19 risk and strong government encouragement.

## Materials and methods

We used data from Time 4 (COVID wave) of The Values Project (https://osf.io/w6uen/). At Time 1, in 2017, The Values Project recruited Australian adults to answer a series of short surveys over time, following a cross-sequential sample design; with a target of 500 respondents in each of 14 age categories from 18 to 75 years of age. Thus, the original sample was intentionally drawn to over-represent older Australians. Despite this, the sample was largely reflective of many socio-demographic characteristics in the Australian population [14]. The study was approved by The University of Western Australia Human Ethics Committee (RA/4/1/8647).

The present study was administered between May 18th and May 29th, 2020 to 1,869 Values Project panel members. Participants provided informed consent online via a check-box after reading information about the study. Of these 1,316 completed all study questions (see S1 Table for completed versus missing sample descriptives): 60% were female, mean age was 58 (SD = 13.20); 31% were parents with children living at home. At the time of the survey, administered prior to the relaxation of social distancing measures in many states, 13% of respondents reported having a household income above or significantly above the Australian average of $120,200. Most had completed high school (M = 13.70 years of education; SD = 3.43).

### Measures

Intention toward the COVID-19 vaccine was measured with one item: *If a COVID-19 vaccine were available today*, *would you get it*? (Yes, Maybe, No). Intention toward the flu injection was measured with one item: *Do you intend to have*, *or have had*, *a flu injection this year*? (Yes, Maybe, No). Disease severity was measured with a 7-item scale [15]. Example items include *I believe that COVID-19 is severe* and *I believe that the COVID-19 threat is significant* (1 = strongly disagree to 7 = strongly agree). Trust in the scientific community was measured with one item, measured on an 11-point scale (0 = no trust at all to 10 = complete trust), added to a longer scale measuring Political Trust in Authorities and Specific Institutions [16]. COVID-19 as a Hoax was measured with a one item scale: *The social media are full of stories telling that the COVID-19 pandemic is a hoax and that all the lockdown measures are a hysteric overreaction*. *Do you believe in these stories*? (YES, I do believe in these stories; NO, I don’t believe in these stories).

### Data analysis

A multinomial logistical regression was used to predict the three vaccination intention responses for a COVID-19 vaccine: yes, maybe, and no. Average marginal effects were estimated, that is the change in probability of each response, for each variable with robust standard errors. In addition, relative risk ratios for each pairing of responses (e.g. maybe vs no), with their 95% confidence interval, were estimated to facilitate comparisons. Regressions were estimated in Stata v16 using the mlogit function. The multinomial logit assumes independence of irrelevant alternatives, which was evaluated, and not rejected, using the mlogtest program. Related, a likelihood ratio test of whether any response categories could be combined (e.g. no with maybe) was rejected [17].

## Results

**Fig 1:**
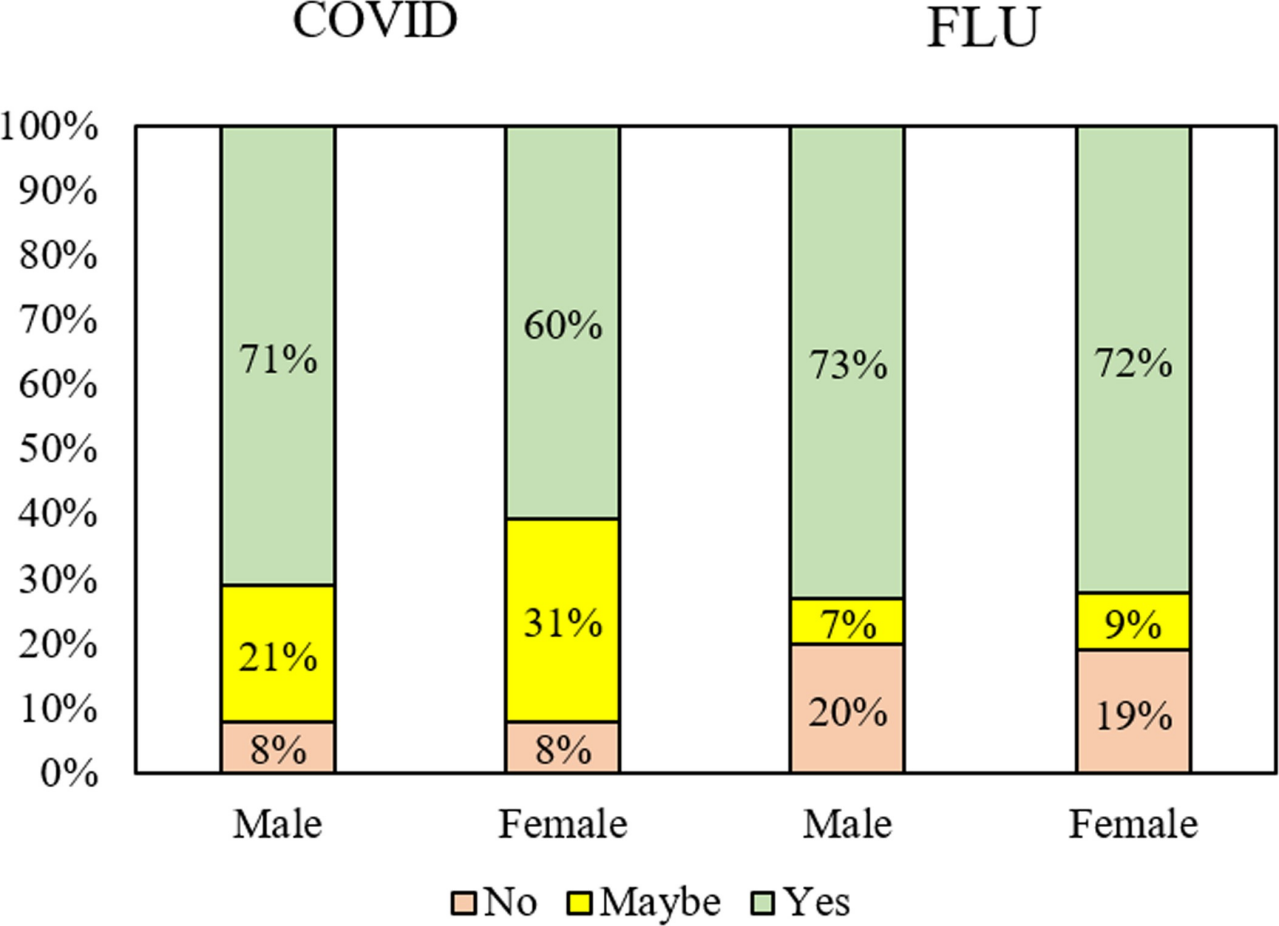
Australians’ intentions to get a COVID-19 vaccine if it was available today. N = 1,316.

We found that 65% respondents were willing and 27% were undecided about whether to get a COVID-19 vaccine if one becomes available (Fig 1). In contrast, intentions regarding influenza vaccination were much more polarized, with fewer people being undecided. We found 69% percent of our respondents having received or intending to have a flu vaccine this year, 21% saying no, and only 10% maybe.

Respondents were more likely (RRR>1) to be in the COVID-19 vaccine ‘maybe’ than ‘yes’ group, if they (a) perceived COVID-19 to be less severe (RRR = 1.54, 95% CI: 1.25 to 1.91), (b) had less trust in science (RRR = 1.35, 95% CI: 1.22 to 1.51), (c) were less willing to have a flu vaccine (RRR = 2.14, 95% CI: 1.81 to 2.55), and (d) were female (RRR = 1.97, 95% CI: 1.47 to 2.64). Compared to the ‘no’ group, respondents were more likely to be in the ‘maybe’ group, if they did not perceive COVID-19 to be a hoax (RRR = 7.70, 95% CI: 3.02 to 19.6). Respondents were less likely (RRR < 1) to be in the ‘maybe’ group relative to the ‘no’ group if they (a) did not perceive COVID-19 to be severe (RRR = 0.74, 95% CI: 0.60 to 0.91), (d) did not have trust in science (RRR = 0.88, 95% CI: 0.79 to 0.97), and (c) were not willing to have the flu vaccine (RRR = 0.49, 95% CI: 0.37 to 0.65). There were no gender differences between the ‘maybe’ and ‘no’ groups (Table 1).

**Table 1:** Multinomial logistic regression of Australian’s intentions to get a COVID-19 vaccine if it was available today.

	Yes	Maybe	No	RRR Maybe vs Yes		RRR No vs Yes		RRR Maybe vs No	
	0.65	0.27	0.08	95% CI		95% CI		95% CI	
(No) Flu injection intention	-0.16***	0.10***	0.06***	2.14***	[1.81,2.55]	0.23***	[0.17,0.30]	0.49***	[0.37,0.65]
	(0.01)	(0.01)	(0.01)						
(Not a) COVID-19 Hoax	0.08	0.04	-0.12***	0.89	[0.35,2.25]	0.12***	[0.04,0.29]	7.70***	[3.02,19.6]
	(0.09)	(0.08)	(0.02)						
(Lack of) Disease severity	-0.03***	0.01	0.02***	1.14**	[1.01,1.29]	1.54***	[1.25,1.91]	0.74***	[0.60,0.91]
	(0.01)	(0.01)	(0.01)						
(Lack of) Trust in science	-0.04***	0.02***	0.01***	1.19***	[1.11,1.27]	1.35***	[1.22,1.51]	0.88***	[0.79,0.97]
	(0.01)	(0.01)	(0.00)						
Female	-0.13***	0.10***	0.03*	1.97***	[1.47,2.64]	2.24**	[1.32,3.86]	0.87	[0.51,1.49]
	(0.03)	(0.02)	(0.01)						
Age	-0.00	0.00	-0.00	1.01	[0.99,1.02]	1.00	[0.98,1.01]	1.01	[0.99,1.03]
	(0.00)	(0.00)	(0.00)						
Children at home	-0.05*	0.05	0.00	1.32	[0.96,1.82]	1.21	[0.71,2.05]	1.10	[0.65,1.86]
	(0.03)	(0.03)	(0.01)						
Above average income	0.06	-0.04	-0.02	0.75	[0.49,1.15]	0.62	[0.28,1.35]	1.21	[0.55,2.65]
	(0.04)	(0.04)	(0.02)						
Education years	0.00	-0.00	-0.00	0.98	[0.94,1.02]	0.99	[0.92,1.06]	0.99	[0.92,1.07]
	(0.00)	(0.00)	(0.00)						

Notes. Outcomes for regression modelling were no, maybe, or yes, and are presented as adjusted odds ratios (95% confidence intervals) in reference to the yes category. To facilitate discussion of what increases the probability of a maybe response, COVID hoax, Disease severity, and Trust in science were reverse coded. N = 1316

* *p* < .05

** *p* < .01

*** *p* < .001. Log-likelihood Base -1103.5, Log-likelihood Full -921.1, Likelihood Ratio Test χ^2^(18) = 260.2, *p* < .001. Nagelkerke Pseudo R- squared = 0.30.

## Discussion

Our study is one of a small but growing number of papers examining COVID-19 vaccine acceptance in Australia, in the lead up to a vaccine becoming available [10, 11]. However, we are the only paper to date showing that the ‘maybe’ group differs significantly from those willing or unwilling to accept a COVID-19 vaccine. Our study had a lower number of ‘yes’ responses than other studies, however it is difficult to compare this number with existing studies, as they employed different methodologies (e.g., collapsing a 4-point agreement scale into two groups [10], or using ‘neutral/no opinion’ as a mid-point rather than ‘maybe’ [9]) and sampled different target populations (e.g., focus on parents, most of whom were younger than our sample [11]).

Our finding that over a quarter (27%) of the sample were undecided about a COVID-19 vaccine suggests that this group will be important to the effective nationwide rollout of the vaccine. Even with high vaccine efficacy [18, 19], high coverage will be required to suppress and eliminate community COVID-19 activity.

One argument for the high proportion of people who remain undecided is that Australia, at the time of the study, had only been mildly affected by COVID-19 by world standards. However, by mid-March, cases were doubling every three days or so, and Australia’s trajectory was being compared to that of the USA, UK, and Italy. Borders and businesses were closing, and governments strictly enforced social distancing, including stay at home orders. Exposure to COVID-19 news was extensive and for many overwhelming [20], and remained so in the lead up to the current survey.

A more compelling argument for the large number of undecideds relates to the tremendous uncertainty about a potential COVID-19 vaccine, including effectiveness, side effects and availability. Uncertainty, combined with the speed of development of a range of potential vaccines, means that knowledge about existing vaccination programs is unlikely to be transferable to COVID-19. This is evident in the fact that our respondents were much clearer about whether they would, or would not, receive an influenza vaccine, with only 10% being undecided. The influenza vaccine is well established and has far less uncertainty factors than COVID-19 vaccination, meaning that individuals are more likely to stay fixed on positions that they have previously developed regarding receipt of influenza vaccines, whether positive or negative. This likely explains why the ‘no’ group for influenza vaccine was much larger than the ‘no’ group for a COVID-19 vaccine, despite Australia experiencing high demand for flu vaccine in 2020. These findings highlight that flu vaccine attitudes and behaviours do not directly translate to COVID-19 vaccine intentions, and hence the need for continuing qualitative and quantitative studies to track attitudes to a new pathogen in uncertain times.

COVID-19 vaccine acceptance research is being conducted in wildly fluctuating contexts, with the virus and policy responses driving dramatic social and economic changes on a continual basis around the world. Since our study in May 2020, the state of Victoria has undergone a gruelling second lockdown, other states such as Western Australia have enjoyed a return to almost-normal life within a hard state border, while after a period of normality South Australia lurched back into lockdown. More recently, states are now reopening borders to each other. Given these divergent experiences, even within the same country, future research should explore their impact on attitudes towards COVID-19 vaccine acceptance, including whether people are more or less certain about accepting the vaccine in scenarios where the virus is out of control.

On the back of announcements regarding multiple vaccines showing both safety and effectiveness in clinical trials [18, 19], and governments touting imminent rollout and introducing vaccines under special permits overseas, we might expect the ‘maybes’ to decrease in number due to greater certainty. However, we did not find this when we asked a subset of 636 participants the same COVID-19 vaccine uptake question on 18–23 November 2020. We found 56% said yes, 31% maybe and 13% no, demonstrating remarkable similarity (in fact, a slight increase) in the proportion of ‘maybes’. This finding comes despite numerous changes regarding available vaccine information, including greater certainty about manufacturers, likely rollout times, and the Australian Government indicating its priority groups to receive the vaccine [21].

Given all of the above, understanding the correlates of the likelihood of acceptance of a COVID-19 vaccine is of crucial importance, particularly due to the large size of the ‘maybe’ group. Our study indicated that belief in disease severity and trust in science are important factors that differ between those who are on the fence and those who would (and would not) accept a COVID-19 vaccine. Greater levels of uncertainty amongst women indicates that messaging oriented towards female audiences should be investigated further; Detoc and colleagues in France found men were more likely to accept a COVID-19 vaccine than women [6], and Goldman et al found that fathers were more likely to accept a COVID-19 vaccine for their children than mothers in a study conducted in the US, Canada, Israel, Japan, Spain and Switzerland [22]. This gender divide is likely to matter, then, not just for accepting vaccines for oneself, but also for one’s children.

Additional research, including qualitative studies, is needed to uncover the beliefs and sentiments of COVID-19 vaccine fence-sitters, as well as the specific kinds of public communication strategies that can convert them to accept the vaccine. A homogenous approach, or one that draws lessons from previous pandemics, will not be sufficient–we need to understand people’s uncertainty around vaccinating against this current pathogen at this current moment. Researchers need to proactively engage with vaccine program developers and governments to seed the understandings gleaned from such studies into policy and programs. Governments, likewise, need to invest in appropriate programs of social research that segment populations into sub-groups and interrogate the values and beliefs that underpin hesitancy and uncertainty.

## Conclusion

Our study has shown that a significant number of people remain undecided about whether or not to get a COVID-19 vaccine, despite the ongoing devastating consequences of the virus for individuals, communities, and economies. This is alarming, given that many governments around the world are formulating policy on the basis that a vaccine for COVID-19 will solve the current crisis. Further research is urgently needed regarding the attitudes, beliefs, and potential mind-changing factors for those who remain undecided about accepting a COVID-19 vaccine.

## Supporting information

S1 Table Descriptive statistics for the final sample and missing respondents. (DOCX)
