# Supplementary material for: Converting the maybes: Crucial for a successful COVID-19 vaccination strategy
Source: PLoS One. 2021 Jan 20;16(1):e0245907. doi: 10.1371/journal.pone.0245907 (PMC7817004; doi:10.1371/journal.pone.0245907)
Supplement: S1 Table — (DOCX) [file pone.0245907.s001.docx]

**S1 Table. Descriptive statistics for the final sample and missing respondents.**

|  | Final Sample | | | Missing Sample | | | Range | |
| --- | --- | --- | --- | --- | --- | --- | --- | --- |
| Variable | *N* | Mean | *SD* | *N* | Mean | *SD* | Min | Max |
| COVID-19 hoax | 1,316 | 0.04 | 0.19 | 277 | 0.05 | 0.22 | 0 | 1 |
| Disease severity | 1,316 | 4.50 | 1.16 | 332 | 4.32 | 1.38 | 1 | 7 |
| Trust in science | 1,316 | 7.55 | 2.07 | 417 | 6.78 | 2.69 | 0 | 10 |
| Female | 1,316 | 0.60 | 0.49 | 553 | 0.66 | 0.47 | 0 | 1 |
| Age | 1,316 | 57.95 | 13.20 | 553 | 55.26 | 12.77 | 20 | 78 |
| Children at home | 1,316 | 0.31 | 0.46 | 332 | 0.37 | 0.48 | 0 | 1 |
| Household Income  (above average) | 1,316 | 0.13 | 0.34 | 202 | 0.17 | 0.38 | 0 | 1 |
| Education (years) | 1,316 | 13.70 | 3.43 | 553 | 13.19 | 3.70 | 0 | 20 |
